# Community-Based ART Service Delivery for Key Populations in Sub-Saharan Africa: Scoping Review of Outcomes Along the Continuum of HIV Care

**DOI:** 10.1007/s10461-021-03568-3

**Published:** 2022-01-17

**Authors:** Olujuwon Ibiloye, Caroline Masquillier, Plang Jwanle, Sara Van Belle, Josefien van Olmen, Lut Lynen, Tom Decroo

**Affiliations:** 1 Institute of Tropical Medicine, Antwerp, Belgium; 2 APIN Public Health Initiatives, Abuja, Nigeria; 3 Research Foundation Flanders, 1000 Brussels, Belgium; 4 University of Antwerp, Antwerp, Belgium

**Keywords:** Key population, Female sex workers, Community-based antiretroviral therapy, HIV

## Abstract

HIV positive key population (KP) often face health system and social barriers to HIV care. KP include sex workers, men who have sex with men, persons who inject drugs, transgender people, and people in prisons and other closed settings. Community-based ART service delivery (CBART) has the potential to increase access to antiretroviral treatment (ART) and enhance retention in care. This scoping review summarized the evidence on the effect of CBART along the continuum of HIV care among KP in sub-Saharan Africa. We searched Pubmed, Web of Science, Google scholar, and NGO websites for articles published between 2010 and April 2020. We synthesized the involvement of KP community members or lay providers in medical task provision, and outcomes along the continuum of HIV care. Of 3,330 records identified, 66 were eligible for full test screening, out of which 12 were included in the review. CBART for KP was provided through: (a) community drop-in-centres, (b) community drop-in-centres plus mobile team, or (c) community-based health centres. KP were engaged as peer educators and they provided services such as community mobilisation activities for HIV testing and ART, ART adherence counselling, and referral for ART initiation. Across the KP-CBART studies, outcomes in terms of ART uptake, adherence to ART, retention in care and viral suppression were at least as good as those obtained for KP attending facility-based care. KP-CBART was as effective as facility-based care. To achieve the UNAIDS 95–95–95 target in sub-Saharan Africa, national programmes should scale-up KP-CBART to complement facility-based care.

**Supplementary Information:**

The online version contains supplementary material available at 10.1007/s10461-021-03568-3.

## Introduction

Key populations (KP) include female sex workers (FSW), men who have sex with men (MSM), transgender people (TG), persons who inject drugs (PWID), and people in prisons and other closed settings [1]. The risk of HIV acquisition among MSM, PWID, sex workers and TG are respectively 22, 22, 21 and 12 times higher than for adults aged 15–49 years [2]. The HIV prevalence among KP varies widely across regions and countries in sub-Saharan African. In 2018, Uganda had the highest HIV prevalence of 85% among FSW while Sudan had the lowest (0.7%) of the continent [3]. In East and Southern Africa, an estimated 54% of sex workers are living with HIV [4]. The HIV prevalence among MSM ranges between 1.2% in Sudan (2018) and 32.9% in Lesotho (2014) [3].

In 2018, KP and their sexual partners accounted for more than fifty percent of all new HIV cases in Sub-Saharan Africa (SSA). In West and Central Africa, KP, their clients and sexual partners accounted for 64% of new infections and for 25% of new HIV infections in East and Southern Africa [4, 5]. Therefore, improving service coverage for KP could avert a significant number of new infections, stabilise and reverse the HIV incidence rates [1]. KP are often stigmatised and discriminated against, which impacts negatively on access to quality HIV care in regular health care facilities. Other factors limiting access of KP to HIV care, including access to antiretroviral treatment (ART), in SSA include: lack of community support, harassment by law enforcement agencies, laws and policies restricting access, criminalization of behaviours or practices and violence [6]. To improve access to community-based antiretroviral therapy service delivery (CBART) models that bring HIV care and treatment closer to the KP community and encourage participation of KP (communities) in HIV service delivery have been advocated for [7]. Also the World Health Organization has recommended decentralization of ART initiation and/or refill and community-based HIV approaches (such as outreach sites, mobile services, home-based services or community-based organizations) to overcome barriers to HIV care [1].

Community-based approaches to HIV care and treatment have proven to be an effective method of reaching people in the general population, particularly for individuals who are hard to reach [8]. These approaches include community-based and venue-based outreach activities, community-based ART initiation and refill, and home-based ART [1, 9]. If adapted to the needs of KP, such programs may also engage KP communities in HIV service delivery. Indeed, participation of PLHIV in chronic lifelong care has been identified as an enabler of service utilisation and adherence to treatment [10]. The CBART model for KP (KP-CBART) is a client-centred approach to ART services that aims to increase utilisation of services and retention in care by organising ART delivery closer to the patients’ home and at the same time strengthen social networks of HIV-positive KP [11].

A recently published review showed the effect of community-based HIV care along the HIV care continuum among FSW. The HIV care continuum “is a public health model that outlines the steps or stages that people with HIV go through from diagnosis to achieving and maintaining viral suppression (a very low or undetectable amount of HIV in the body” [12]. However, this review only focussed on FSW, included studies regardless of the level of involvement of FSW in care provision, and included studies regardless of whether ART was provided within the communities. Moreover, the modalities used for KP-CBART were not summarized [13]. In our review, we explored the effect of CBART along the continuum of HIV care on clinical outcomes among KP in sub-Saharan Africa. We collected data on community-based HIV care for any type of KP, and only included studies if KP were involved in care delivery along the HIV care continuum, and if care delivery also included ART.

We described the KP-CBART model used, including the type of services provided and the type of KP among beneficiaries, the roles of KP and other key stakeholders in HIV service delivery, and the outcomes of KP-CBART interventions along the continuum of HIV care and treatment (uptake of HIV testing services, linkage to ART, retention, and viral suppression). We also summarized the predictors of retention among KP receiving care in KP -CBART models.

## Methods

This is a scoping review of the evidence on the effect of community-based ART service delivery on clinical outcomes along the continuum of HIV care for KP in SSA countries. Outcomes along the continuum of HIV care include HIV testing uptake, diagnosis of an HIV infection, linkage to care, medication adherence, and ART outcomes (viral suppression and retention [or the opposite, attrition, including those who died or were lost to follow-up (LTFU)]. The framework was developed as recommended by Arksey and O’Malley [14]. This framework helps to clarify when a scoping study is the appropriate method to adopt for evidence synthesis and how to go about the review [14]. The methodology used in this review is coherent with the Preferred Reporting Items for Systematic reviews and extension for Scoping Reviews (PRISMA-ScR) [15].

### Eligibility Criteria

Articles were included in the review if they:

- presented data on community-based (delivered from a community venue) or community-led (involvement of KP community members or lay providers in medical HIV service provision) ART delivery programme for KP (FSW, MSM, PWID and/or TG) in Sub-Saharan Africa, and
- reported data on one or more ART outcomes.

Papers published before 2010 were not considered, as community-based approaches to HIV care and ART delivery became popular after this time [16]. Only articles that were written and published in English were included.

### Information Sources

We searched and extracted articles between April 2020 and May 2020 from the Medline database (through Pubmed). Web of Science was searched in September 2021. The grey literature (Google Scholar) and Websites of World Health Organization (WHO), International AIDS Society (IAS) and other international HIV agencies ( non-governmental organizations) were also searched. Finally, reference lists of retrieved studies were searched.

### Search

Two researchers (IO and TD) conducted the literature review. The search string was constructed using the PICO approach (Table 1). Terms were entered as Medical subheading terms (MeSH) or free text string as shown in Table 1.

**Table 1:** Search strategy using the PICO approach

Sources		Search string for Pubmed
Medline (Pubmed), Web of Science*, & Google Scholar	Population	(“sex workers” [MeSH] or “FSW” or “MSM” or “men who have sex with men” or “transgenders” or “persons who inject drugs” or “PWID” or “IVDU”) AND “Africa”
Websites of KP organizations	Intervention	“Community” or “peer”[All field] or “home”) AND (“HIV” [MeSH]” or “antiretroviral therapy” or “ART” or “HAART”)
	Comparison	Not applicable
Reference list of retrieved citations	Outcomes	“linkage” or “retention” or “attrition” or “viral suppression” or “treatment outcome” [MeSH]

*Search string for Web of Science is in Supplementary Table 2

### Selection of Sources of Evidence

After conducting the search, duplicates were identified and removed using Mendeley reference software. Thereafter, titles and abstracts of retrieved studies were screened. Only studies that presented original data on the topic of interest were retrieved. Then the full text of retrieved studies was read and the inclusion criteria were applied.

### Data Charting Process

Extracted data were encoded in a data collection tool (Excel sheet), conceived and tested before the start of the review (see the attached Supplementary File 1 for the data extraction template).

### Data Collection and Synthesis

We collected thematic data on KP-CBART delivery, such as type of CBART model, intervention components, characteristics of lay providers (including KP) and their roles, and capacity building (training) of care providers.

We collected quantitative data on the study title, author, publication year, study period, study location, study design, study setting, sample size, participant characteristics (e.g. HIV status, KP type), and continuum of HIV care outcomes. Outcomes included uptake of HIV testing, HIV testing positivity among those tested, linkage to HIV care among those tested positive, ART uptake among those linked to care, medication adherence, clinical outcomes among those on ART, including retention (or the opposite: attrition, which usually was defined as the sum of deaths and those LTFU), and viral suppression. We also collected data on the definitions used for these outcomes. Finally, data were collected on factors associated with retention or attrition, as reported in the studies. The collected data were summarized in Tables 2, 3, 4, 5, 6, 7 and 8).

**Table 2:** Characteristics of studies documenting CBART among key populations

Study (author & date)	Setting	Study design	Study population	Intervention (CBART)	^§^Control	Follow-up period	Reported outcomes				
							HIV testing uptake	ART uptake	ART Adherence	Attrition	VLS
Kerrigan et al. 2019 [17]	Tanzania	Community RCT	FSW	Community drop-in center	Standard care: HIV services through government facilities and NGO-led facilities	18 months	NA	Yes	Yes	Yes	Yes
Cowan et al. 2018 [18]	Zimbabwe	Cluster RCT	FSW	Community drop-in center	Standard care: referral for ART and offer HIV testing on demand	2 year	Yes	Yes	NA	NA	Yes
Napierala et al. 2018 [20]	Zimbabwe	Survey	FSW	Community-drop in centre	NA	2 months	NA	Yes	NA	NA	Yes
W. Tun et al. 2019 [21]	Tanzania	Prospective cohort study	FSW	Community drop-in center with mobile team	Standard care: community-based HCT without ART & mobile team	1 year	NA	Yes	Yes	Yes	NA
Ibiloye et al. 2018 [22]	Nigeria	Retrospective cohort	MSM, FSW, PWID	Community drop-in center with mobile team	NA	1 year	Yes	Yes	Yes	Yes	Yes
Olawore et al. 2020 [23]	Cote d’Ivoire	Retrospective cohort	FSW	Enhanced peer outreach within the LINKAGES’ project (community drop-in centre)	Traditional peer outreach	6 months	Yes	Yes	NA	NA	NA
LINKAGES 2017 [24]	Multi-country (Ivory Coast, Democratic Republic of the Congo (DRC), Malawi, and South Sudan)	Programme report based on program data (Cote d’Ivoire)	MSM, FSW,	Community drop-in center (all sites) *Plus* enhanced peer outreach approach (in Cote d’Ivoire)	Traditional peer outreach	3 months	Yes	Yes	NA	NA	NA
Ramadhani et al. 2018 [25]	Nigeria	Prospective cohort study	MSM	Community-based health centre	NA	4 year	Yes	Yes	NA	Yes	Yes
Graham et al. 2018 [19]	Kenya	RCT	GBMSM	Community-based health centre (NSC & peer support)	Standard adherence counselling	2 years	NA	NA	Yes	Yes	Yes
Kayode et al. 2020 [26]	Nigeria	Prospective cohort	MSM	Community- based health centre	NA	18 months	NA	NA	NA	Yes	Yes
Charurat et al. 2015 [27]	Nigeria	Prospective cohort	MSM	Community-based health centre	NA	12 months	NA	Yes	NA	Yes	NA
Diallo et al. 2020 [28]	Benin	Prospective cohort	FSW	Community-based health centre	NA	24 months	NA	NA	Yes	Yes	Yes

^§^Conventional care: diverse across settings

*NA* not available, *RCT* randomised controlled trial, *FSW* female sex worker, *MSM* men who have sex with men, *PWID* persons who inject drug, *VLS* viral load suppression, *NSC* next step counselling, *HCT-HIV* counselling and testing, *GBMSM* gay, bisexual, and other men who have sex with men, *ART* antiretroviral therapy

**Table 3:** Characteristics of the described KP-CBART interventions

KP-CBART intervention	Study	CBART interventions	Care providers and their roles in service delivery	Training	Inclusion criteria	Frequency of visit
Community drop-in-centre [17, 18]	Kerrigan et al. 2019 [17]	- Community-led drop-in centre - Mobilisation - Venue-based peer education - Condom distribution - HIV testing, ART - Peer service navigation - SMS reminders, - Provider sensitivity training - Support group meetings	Peer educators: - Community mobilisation - Condom distribution - Accompanied referral - Venue based peer education - Support HIV testing and counselling and ART HIV clinical care providers: - Syndromic management of STI - ART initiation	Provider sensitisation training Workshops on topics such as stigma, discrimination, and GBV; family planning; HIV/sexually transmitted infection prevention including condom negotiation; ART adherence; financial security; and sex worker rights and community mobilization strategies	Women aged 18 years or older who reported exchanging sex for money	Every month
	Cowan et al. 2018 [18]	Drop-in-centres plus Adherence sisters program (for those on ART & PrEP) Onsite ART & PrEP initiation	Health professionals: - ART & PrEP initiation Peer educators: - Community mobilisation - Coordinate adherence sisters program for clients on ART and PrEP Adherence supporters (Sister): - Act as treatment partner - Accompany clients to support group meetings & DIC	NA	FSW aged 18 years or older	Every month
	Napierala et al. 2018 [20]	Community drop-in centre: survey of the Sisters Antiretroviral therapy Programme for FSW	The same as Cowan et al. 2018	NA	18 years and older and currently working as FSW	NA
Community drop-in center with mobile team [19, 21]	W Tun et al. 2019 [21]	CBHTC and ART mobile and home-based platform (CBHTC + team service model)	Community-based health services team - Consists of 1 clinician, 2 nurses, 3 peer educators - HIV counselling and testing - ART initiation - ART adherence counselling Peer educators: enrollment & ART initiation Non-clinical staff: - HTC, STI screening - Escorted referrals of HIV positive clients to health facilities - Condom promotion and provision - Referrals for cases of GBV, TB screening, and alcohol and drug screening	Training on assessing the client’s readiness and ART initiation, ART delivery, ART adherence and protocol on referral for advanced treatment in comprehensive treatment centre	FSW aged 18 years and above Clinically stable (WHO 1 & 2)	Every month
	Ibiloye et al. 2018 [22]	Community-based health centre, Drop-in-centres & ART mobile/outreach (OSS model)	Community facilitators: - Community mobilisation and HIV testing & counselling, - Medication adherence counselling and tracking of LTFU patients - ART refill ART clinic/outreach team: - Consists of Doctor, Nurse, Laboratory scientist and community facilitators - ART outreaches to hotspots - HTS, onsite ART initiation, STI treatment	ART training, KP sensitization training	KP aged 18 years or older	Every month
	Olawore et al. 2020 [23]	Community based outreach (Enhanced peer outreach)	Peer outreach workers (POW) - Are members of KP - Recruit peer mobilsers - Deliver HIV prevention, testing, and ART adherence support services Peer mobilisers (PM) -Reach out to social and sexual networks -Encourage peers for HIV testing, treatment, and other services	POW are trained by local CBOs and implementing partner PM are not formally trained, but they are familiarized with EPO process, participants selection, and coupons distributions	Member of the KP and not previously engaged with an HIV program	
	LINKAGES 2017 [24]	Drop-in-centres and community based outreach (enhanced peer outreach approach)	Peer educators: - Social network HIV testing - Referral to DIC or health facilities for ART initiation - Information, education and counselling - Condoms and lubricants provision	Workshop on microplanning tools, an individual peer plan, a 90–90–90 framework analysis, and opportunity gap analysis	FSW, MSM	NA
Community-based health centre [22]	Ramadhani et al. 2018 [25]	Community-based centre: provision of MSM friendly health care services	Health care professionals: - Provide comprehensive HIV services to MSM KP opinion leaders: - Recruitment of study participants (respondent driven sampling)	NA	MSM, age ≥ 16 years	Monthly
	Graham et al. 2018 [19]	Community-based centre for GBMSM	Research counsellors and clinical officers - Provide Next Step Counselling approach using motivational interviewing technique Peer educators - Provide positive role models - Offer counseling and education - Support medication adherence and HIV status disclosure - Emphasize the importance of pill-taking, send appointment reminders, and tracing of participants who missed visits	One week of peer education by the Kenyan National AIDS and STD Control Pro-gramme	MSM, age ≥ 18 years	Monthly
	Kayode et al. 2020 [26]	Community-based centre (co-located with CBO) for MSM	Peer mobilisers -Clients referral through respondent driven sampling (provide referral coupons) Advocacy group and health care professionals -Education about safer sex practices -Distribution of condoms and lubricants -Diagnosis and treatment of HIV and other STIs	Sensitization training to meet social, legal and sexual health needs of study participants	Age ≥ 16 years, MSM,and a valid referral coupon	
	Charurat et al. 2015 [27]	Community-based centre (CBO supported community-based clinic dedicated to MSM)	Health professionals and lay health workers (*counselors, nurse case manager, pharmacist, physician, palliative care officer, and laboratory scientist*) -Client recruitment:respondent driven sampling (Community-based convenience sampling) -Offer clinical and laboratory monitoring -ART initiation and refill	-MSM sensitivity training (culturally sensitive service delivery) -Standard training in HIV/STI management	MSM ≥ 16 years old	
	Diallo et al. 2020 [28]	Community-based centre (research HIV & STI clinic dedicated to FSW)	Health professionals -ART and PrEp initiation -STI screening and treatment -clinical examinations -laboratory monitoring (CD4 & VL test) Field workers - Clients tracking and documentation of tracking outcomes	Speciffic education on adherence	FSW- 18 years and older	

*NA* not available, *STI* sexually transmitted infections, *STD* sexually transmitted diseases, *GBV*gender based violence, *PrEP* pre-exposure prophylaxis, *CBHTC* community-based HIV test and counselling, *ART* antiretroviral therapy, *VL* viral load, *CBO* community-based organization, *MSM* men who have sex with men, *FSW* female sex workers, *DIC* drop-in centre, *LTFU* lost to follow up

**Table 4:** HIV testing and linkage to HIV care among KP in CBART models

KP-CBART intervention	Study	Study population/size	Indicator	Intervention (KP-CBART)		^§^Control		Effect
				Baseline	Follow-up	Baseline	Follow-up	
Community drop-in-centre	Kerrigan et al. 2019 [17]	171 HIV +ve FSWs Intervention: 91 Control: 80	Linkage to HIV care	28.6% (26/91)	79.1% (72/91) *p* < 0.001 (before-After)^$^	18.8% (15/80)	55.0% (44/80) *p* < 0.001 (before-After)^$^	RR = 1.44, *p* = 0.02
	Cowan et al. 2018 [18]	Intervention arm BL- 1317 FSWs; FU- 1397 FSWs Control arm: BL- 1252; FU-1393	HIV testing positivity (Based on survey at baseline and at the end of assessment period)	40.4% (1052/2606)	79.5% (669/829) (95% CI 63.5–88)	47.4% (546/1151)	78.4% (695/869) (95% CI 65.1–86.2)	Adjusted risk difference (aRD) = 0.2% (95% CI -8.8 to 2.5) p = 0.95
	Napierala et al. 2018 [20]	FSW	HIV prevalence (% of HIV positive FSW)	NA	NA	NA	Young FSW = 35% Older FSW = 67% (p = 0.01)	NA
Community drop-in-centre with mobile team	W Tun et al. 2019 [21]	CBART arm = 256 FBART arm = 253	Linkage to care	NA	100% (256/256)	NA	72.7% (184/253)	*p* < 0.001^$^
	Ibiloye et al. 2018 [22]	KP and sexual partners- 710	HIV testing positivity	NA	6.1% (935/15274)	NA	NA	NA
	Olawore et al. 2020 [23]	9761 FSW reached EPOA = 2509 Routine peer outreach = 7252	HIV case finding	NA	10.7% (269/2507)	NA	6.8% (429/6344)	X^2^ = 32.3, p = 0.001
			Linkage to treatment	NA	95.9% (258/269)	NA	71.3% (306/429)	X^2^ = 64.4, p = 0.001
	LINKAGES 2017 [24]	FSW- 3476, MSM-714 (Cote d’Ivoire)	HIV testing positivity	NA	FSW = 5.6%; MSM = 15.4%	NA	FSW = 1.7%; MSM = 5.9%	NA
			% of HIV testing in the project through EPOA	NA	MSM = 37%; FSW = 31%	NA	NA	NA
			% of HIV diagnosis in the project through EPOA	NA	MSM = 65%; FSW = 54%	NA	NA	NA
Community-based health centre	Ramadhani et al. 2018 [25]	MSM-1506	Proportion of patients offered HIV test	NA	78.2% (1178/1506)	NA	NA	NA
			HIV testing positivity	NA		31.3% (369/1178)	NA	NA
	Graham et al. [19]		NA	NA	NA	NA	NA	NA
	Kayode et al. 2020 [26]	MSM	NA	NA	NA	NA	NA	NA
	Diallo et al. 2020 [28]	FSW—107	NA	NA	NA	NA	NA	NA
	Charurat et al. 2015 [27]		HIV testing positivity	NA	31.3% (369/1178)	NA	NA	NA

^$^The original study reported a p-value without a value for the statistical test

^§^Conventional care: diverse across settings

*EPOA* enhanced peer outreach approach, *BL* baseline, *FU* follow up or end of assessment period, *NA* not available, *FBART* facility-based ART, *CBART* community based ART, *FSW* female sex workers, *MSM* men who have sex with men

**Table 5:** Uptake of ART among KP in CBART models

KP-CBART model	Study	Study population/size	Indicator	Intervention (CBART)		^§^Control		Effect
				Baseline	Follow-up	Baseline	Follow-up	
Community drop-in-centre	Kerrigan et al. 2019 [17]	171 HIV +ve FSWs Intervention: 91 Control: 80	Ever on ART	28.0% (26/91)	82.4% (75/91) *p* < 0.001 (before-After)^$^	18.8%(15/80)	67.5% (54/80) *p* < 0.001 (before-After)^$^	RR = 1.22, *p* = 0.03
	Cowan et al. 2018 [18]	Intervention arm BL- 1317 FSWs; FU- 1397 FSWs Control arm: BL- 1252; FU-1393	Reported being HIV positive and taking ART	NA	86.3% (95% CI 78.7–96.0) 594/669	NA	83.0% (95% CI 72.4–89.8) 580/695	aRD = 3.4% (-2.9 to 9.7), p = 0.22
	Napierala et al. 2018 [20],	FSW	ART uptake	NA	NA	NA	< 25 years old = 55% = / > 25 years old = 68% (p = 0.06)^$^	NA
Community drop-in-centre and mobile team	W. Tun et al. 2019 [21]	CBART arm = 256 FBART arm = 253	Initiated on ART	NA	100% (256/256)	NA	71.5% (181/253)	*p* = 0.04^$^
	Ibiloye et al. 2018 [22]	KP and sexual partners- 710	Initiated on ART	NA	77.4% (724/935)	NA	NA	NA
	Olawore et al. 2020 [23]	FSW	ART initiation	NA	78.8% (212/269)	NA	73.3% (315/429)	X^2^ = 2.6, p = 0.11
	LINKAGES 2017 [24]	FSW- 3476, MSM-714 (Cote d’Ivoire)	Initiated on ART (% contribution of EPOA to overall project result)	NA	MSM = 83% FSW = 54%	NA	NA	NA
Community-based health centre	Ramadhani et al. 2018 [25]	MSM-1506	% of HIV positive patients initiated on ART	NA	50.1% (188/369)	NA	NA	NA
	Graham et al. 2018 [19]	GBMSM	NA	NA	NA	NA	NA	NA
	Kayode et al. 2020 [26]	MSM	ART initiation	NA	NA	NA	NA	NA
	Charurat et al. 2015 [27]	MSM- 706	ART initiation	NA	NA	NA	54.7% (70/128)	NA
	Diallo et al. 2020 [28]	FSW- 111	ART Initiation	NA	NA	NA	96.3% (107/111)	NA

^$^The original study reported a p-value without a value for the statistical test

^§^Conventional care: diverse across settings

*EPO*A enhanced peer outreach approach, *BL* baseline, *FU* follow up or end of assessment period, *aRD* adjusted risk difference, *FBART* facility-based ART, *CBART* community based ART

**Table 6:** Adherence to ART among KP in CBART models

KP-CBART model	Study	Study population/size	Measure of ART adherence	Intervention (CBART)		^§^Control		Effect
				Baseline	Follow-up	Baseline	Follow-up	
Community drop-in-centre	Kerrigan et al. 2019 [17]	171 HIV +ve FSWs Intervention: 91 Control: 80	Self-reported adherence to ART in the last 4 days	25.3% (23/91)	71.4% (65/91) *p* < 0.001 (before-After)^$^	11.3% (9/80)	46.2% (37/80) *p* < 0.001 (before-After)^$^	Compare at Follow-up: RR = 1.54, *p* = 0.002
	Cowan et al. 2018 [18]	Intervention arm BL- 1317 FSWs; FU- 1397 FSWs Control arm: BL- 1252; FU-1393	NA	NA	NA	NA	NA	NA
	Napierala et al. 2018 [20]		100% ART Adherence (self report)	NA	NA	NA	Young FSW = 83% Older FSW = 88%	NA
Community drop-in-centre and mobile team	W Tun et al. 2019 [21]	CBART arm = 256 FBART arm = 253	Stop taking ART for more than 30 days	NA	0.9% (2/214)	NA	5.7% (9/159)	*p* = 0.008
	Ibiloye et al. 2018 [22]	KP and sexual partners- 710	Good medication adherence- not missing more than 3 doses of ART/month	NA	87.3% (505/578)	NA	NA	NA
	Olawore et al. 2020 [23]	FSW	NA	NA	NA	NA	NA	NA
	LINKAGES 2017 [24]	FSW- 3476, MSM-714 (Cote d’Ivoire)	NA	NA	NA	NA	NA	NA
Community-based health centre	Ramadhani et al. 2018 [25]	MSM-1506	NA	NA	NA	NA	NA	NA
	Graham et al. 2018 [19]	GBMSM Intervention arm Control arm	Proportion of participants with post-intervention VAS adherence ≥ 80% (i.e.month 1-month 6)	NA	Month 1–6: 59–75%	NA	Month 1–6: 70–82%	aOR 1.76, 95% CI 0.70–4.41, Z = 1.21, p = 0.2
	Kayode et al. 2020 [26]	MSM	Visit adherence: rate of visits completed per three-month interval	NA	HIV −ve: 0.51 (95% CI 0.49 to 0.54) HIV +ve: 0.72 (95% CI 0.69 to 0.74)	NA	NA	aRR = 0.80 (95% CI 0.75–0.85)
	Charurat et al. 2015 [27]	MSM	NA	NA	NA	NA	NA	NA
	Diallo et al. 2020 [28]	FSW	Prevalence of virally suppressed FSWs according to self-reported adherence: < 3 pills missed/month = high adherence (≥ 90%),	NA	NA	NA	High adherence: 83.2% (129/155) PR = 1.4, 95% CI 1–2. p = 0.04	NA

^$^The original study reported a p-value without a value for the statistical test

^§^Conventional care: diverse across settings

*NA* not available, *FBART* facility based ART, *CBART* community based ART, *GBMSM* gay, bisexual, and other men who have sex with men, *RR* relative risk, *aRR* adjusted relative risk, *aOR* adjusted odd ratio

**Table 7:** Virological outcomes among HIV positive KP in CBART programs

KP-CBART model	Study	Study population/size	Indicator (VLS)	Intervention (CBART)		^§^Control		P-value
				Baseline	Follow-up	Baseline	Follow-up	
Community drop-in-centre	Kerrigan et al. 2019 [17]	171 HIV +ve FSWs	VL < 400 copies/ml	40.0% (36/91)	50.6% (46/91)	35.8% (28/80)	47.4% (36/80)	RR = 1.05, p = 0.742
		Intervention: 91			p < 0.154^$^		p < 0.149^$^	
		Control: 80			(before-After)		(before-After)	
	Cowan et al. 2018 [18]	Intervention arm	VL < 1000 copies/ml	NA	72.0%	NA	67.50%	Adjusted risk difference- 5.3% (− 4.0% to 14.6%)
		BL- 1317; FU- 1397 FSWs			(95% CI 63.8–86.8)		(95% CI 61.4–73.1) 590/869	p = 0.20
					588/828			
		Control arm:						
		BL- 1252; FU-1393						
	Napierala et al. 2018 [29]		Viral suppression, was defined as a VL of < 1000 copies per milliliter	NA	NA	FSW < 25 years: VLS = 62%	62% of younger FSWs reporting ART use had a VL < 1000 copies/ml, compared with 79% in older FSWs (*p* = 0.06)^$^	
						FSW ≥ 25 years: VLS- 79%		
						(p = 0.09)^$^		
Community drop-in-centre and mobile team	W Tun et al. 2019 [21]	NA	NA	NA	NA	NA	NA	NA
	Ibiloye et al. 2018 [22]	KP and sexual partners- 710	VL < 1000 copies/ml	NA	88% (157/178)	NA	NA	NA
	Olawore et al. 2020 [25]	NA	NA	NA	NA	NA	NA	NA
	LINKAGES 2017 [24]	NA	NA	NA	NA	NA	NA	NA
Community-based health centre	Ramadhani et al. 2018 [25]	MSM-1506	VLS < 1000 copies/ml	NA	70.6% (96/136)	NA	NA	NA
	Graham et al. 2018 [19]	GBMSM	VLS (≤ 40 copies/mL) at baseline, month 3, and month 6		Baseline—43%		Baseline—62%	aOR 6.07 (1.40–26.2) Z = 1.21, p = 0.23Z = 2.41, p = 0.02
					Month 3–78%		Month 3–100%	
					Month 6–78%		Month 6–96%	
	Kayode et al. 2020 [27]	MSM	NA	NA	NA	NA	NA	NA
	Charurat et al. 2015 [28]	MSM	Undetectable VL < 200 copies/ml at 6 months on ART			NA	80.4% (37/46)	
	Diallo et al. 2020 [30]	FSW	VLS < 1000 copies/ml	NA	NA	Baseline: 20.5% (22/107)	At 6 months: 73.1%	NA
							At 12 months: 84.8%	
							At 24 months: 88.2%	

^$^The original study reported a p-value without a value for the statistical test

^§^Conventional care: diverse across settings, key population

*VLS* viral load suppression, *VL* viral load, *NA* not available, *BL* baseline, *FU* follow up, *FBART* facility-based ART, *CBART* community based ART, *MSM* men who have sex with men, *FSW* female sex workers.

**Table 8:** Retention rate and factors associated with retention in care

KP-CBART model	Study	Outcome definitions	Follow-up period	Retention		Factors associated with retention-in-care
				Intervention (CBART)	^§^Control	
Community drop-in-centre	Kerrigan et al. 2019 [17]	Retention: proportion of patients that are receiving care at 18 months of follow-up LTFU: is the opposite of retention. Patient who has stopped receiving care at 18 months of follow-up ART current: Number of patients who are currently receiving antiretroviral therapy	18 months	Overall retention rate among study participants (both HIV positive and negative FSW)—81.5% (404/496) ART current: BL- 28.6% (26/91) FU—81.3% (74/91) *p* < 0.001 (before-After) Compare at follow-up: RR = 1.27, *p* = 0.01	ART current: BL—17.5% (14/80) FU—63.8% (51/80) *p* < 0.001 (before-After)^$^	Retention was correlated with older age and level of education (measure of effect not shown in the paper) LTFU was correlated with possession of mobile phone and having worked in the venue for less than 6 months Dose response analysis: Both medium and highest level of exposure to intervention were associated with engagement in HIV care (within the last 6 months)-(RR-1.85; 95% CI 1.12–3.07) and RR: 2.15; 95% CI 1.08–2.71) and current on ART(RR:1.71; 95% CI 1.24–3.02)
	Cowan et al. 2018 [18]	NA		NA	NA	NA
	Napierala et al. 2018 [20]	NA	NA	NA	NA	NA
Community drop-in-centre and mobile team	W Tun et al. 2019 [21]	LTFU: patient who is not receiving any intervention or standard care at 6-month of follow up. Patients who are LTFU include those that cannot be reached, transferred out and dead	6 months	Of the 309 enrolled in care, 256 (82.8%) completed 6 months follow-up visit Current on ART at the 6-month visit -100.0% (254/254) LTFU-53: Died- 3 Discontinue- 5 Not reachable-42 Transferred out- 3	Of the 308 enrolled in care, 253 (82.1%) completed 6 months follow-up visit Current on ART at the 6-month visit -95.0% (171/180) LTFU-55: Died- 1 Discontinue- 3 Not reachable-37 Wrong telephone no. 14	No significant differences in terms of age, marital status, education, number of living children, income from sex work, average monthly income, and traveling outside the region to sell sex among those LTFU and those retained in the study When stratified by study arm: -There were no significant differences between the LTFU and those who remained in the intervention arm -In the comparison arm, LTFU were slightly younger and less likely to have travelled outside of the region for sex work in the past 6 months
	Ibiloye et al. 2018 [22]	Retention in care on ART: proportion of patients that are retained in care at 6 months of ART, among those who started ART LTFU are those lost from the programme for more than 2 months since their last appointment	7 months	13.9% (99/710) discontinued ART after their first visit After a median follow-up time of 7 months on ART, 73.2% (520/710) of patients were retained, 23.4% (166/710) were LTFU, and 3.4% (24/710) were dead	NA	Factors associated with attrition are lack of formal education (aHR 1.8; 95% CI 1.3–2.6) and unemployment (aHR 1.8; 95% CI 1.2–2.6)
	Olawore et al. 2020 [23]	NA	NA	NA	NA	NA
	LINKAGES 2017 [24]	NA	NA	NA	NA	NA
Community-based health centre	Ramadhani et al.,2018 [22]	Retention: Proportion of patients who initiated ART that remained on ART at 6-months of follow up	6 months	72.3% (136/188) completed six months of ART	NA	NA
	Graham et al. 2018 [19]	Retention: proportion of patients that are retained in care at 6 months of follow-up	6 months	Retention—85%	Retention—85% Χ2 = 0.0013, p = 1.00^$^	Attrition did not differ by study arm More men who had only male sex partners were lost to follow-up than men with both male and female partners [6 of 15 (40.0%) vs. 3 of 45 (6.7%), Χ2 = 9.80, p = 0.005] More men who had participated in the study for < 12 months were lost to follow-up than those who had participated for ≥ 12 months [7 of 25 (28.0%) vs. 2 of 35 (5.7%), Χ2 = 5.68, p = 0.03] Men with lower CD4 counts at baseline were also more likely to be lost to follow-up (median CD4 count 267 vs. 510, Z = − 2.50, p = 0.01^$^
	Kayode et al. 2020 [26]	LTFU was defined as not presenting for an expected visit in the past 180 days	18 months	LTFU rate among HIV positive = 24% (359/808) HIV +ve vs HIV −ve MSM: aHR- 1.72 (95% CI 1.49–2.0) p < 0.01	NA	Retention was suboptimal for both MSM and TGW After controlling for other factors, LTFU was less common among participants living with HIV or other STIs and more common among those who did not own a cell phone, sold sex and had never undergone HIV testing prior to enrolment
	Charurat et al. 2015 [27]	LTFU was defined as not having a visit within 3 months from the last visit	18 months	10% at 18 months since enroment	NA	Being engaged in TasP (HR 0.08, p < 0.001) and on ART (HR 0.17, p < 0.001) were associated with decreased risk of LTFU
	Diallo et al. 2020 [28]	Rate of drop out from the study	12–24 months	40.2% dropped out between recruitment and end of study	NA	Reason for dropped out is mainly mobility of sex workers to change work setting; seeking clients in other cities or countries

^$^The original study reported a p-value without a value for the statistical test,

^§^Conventional care: diverse across settings,

*BL* baseline, *FU* follow up or end of assessment period, *NA* not available, *LTFU* lost to follow up, *aHR* adjusted hazard risk, *HR* hazard risk, *TasP* treatment as prevention, *TGW* transgender women

## Results

**Fig. 1:**
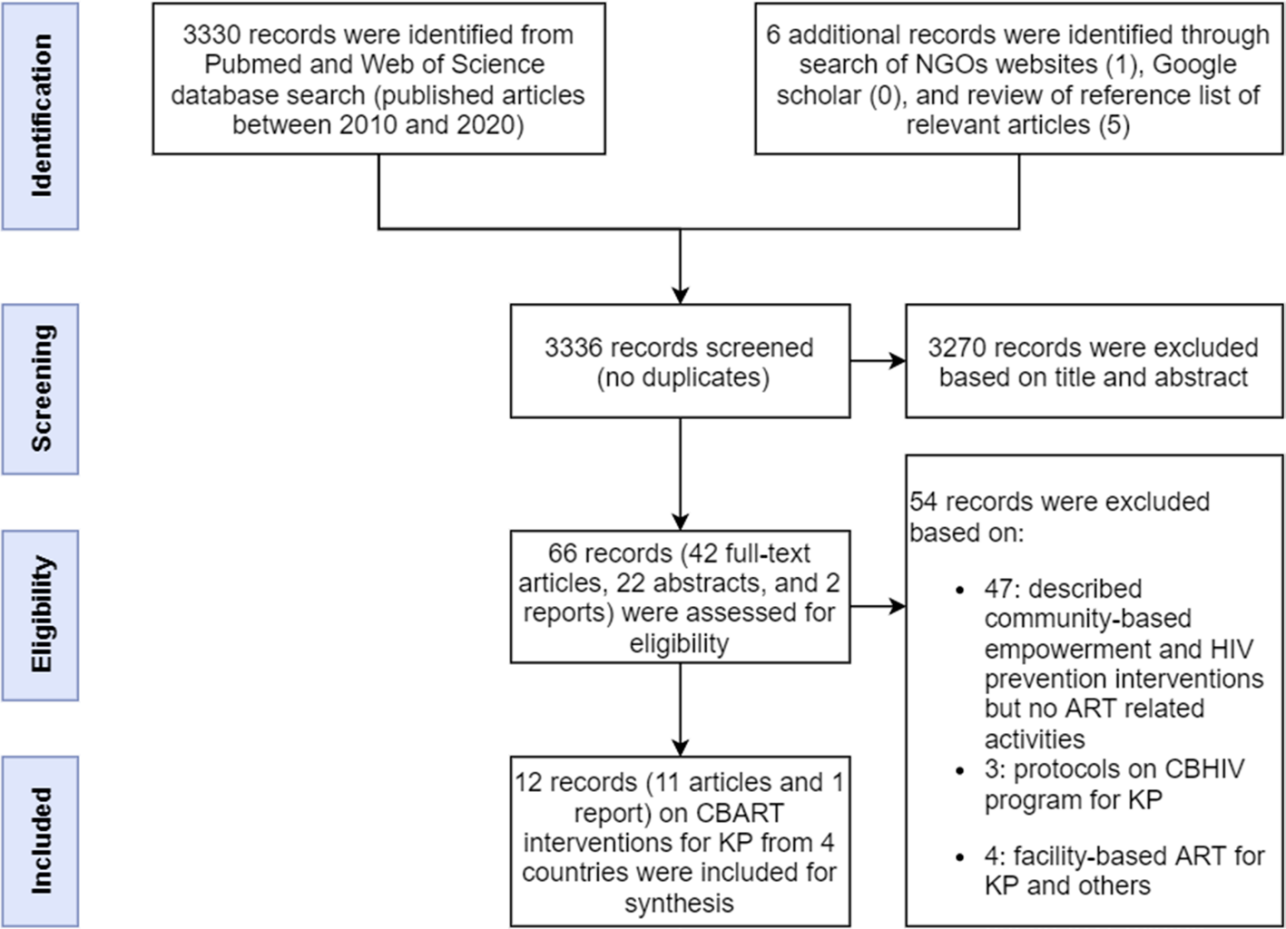
Prisma diagram of literature search for KP-CBART study and selection process

The selection of articles is shown in Fig. 1. The database search identified 3330 published articles in Pubmed (3033) and Web of Science (297). Additional search on Google scholar did not result in papers not yet retrieved from Pubmed. One paper was identified on a non-governmental organizations’ website. From the reference lists of relevant articles we retrieved five additional papers. The titles and abstracts of identified papers were screened to remove duplicates and to exclude papers that did not show original data or did not provide data on community-based service delivery for key populations (See prisma). The full text of the remaining 66 selected articles was read. After applying the inclusion criteria, 11 articles and 1 report were included in the review.

The twelve papers described CBART programs for KP in 6 sub-Saharan African countries. The study characteristics are summarized in Table 2. Three of the studies were randomised controlled trials (RCT) [17–19]. One RCT enrolled FSW and was randomised at the community level [17] and the other two randomized at the level of care delivery sites studied FSW [18] and MSM [19]. The other nine studies were observational studies, including five prospective studies, two retrospective studies, one report showing a retrospective data analysis, and a survey [20–28]. Six observational studies targeted FSW. One observational study [22] and one report on multi-country community-based HIV program in Ivory Coast, Malawi, South Sudan and Democratic Republic of Congo (DRC) targeted both MSM and FSW [24]. Three observational studies were designed for MSM only [25–27].

### Thematic Data on KP-CBART Delivery

#### Community-Based ART (CBART) Models

ART services were provided to KP through either a community Drop-in-Centre (DIC), a community DIC with a mobile team, or a community-based health facility (Tables 2 and 3). One site also offered enhanced peer outreach next to a DIC. What differentiates these models from each other are the locations of service delivery, the roles of care providers in ART delivery, and the package of HIV services offered to KP.

A DIC aims at being safe for members of KP to meet, make friends and develop a sense of community [24]. To enhance accessibility and acceptability for KP, DIC were located in close proximity to the hotspots (a location in the community where members of KP gather or meet) and were run by KP themselves. Operation hours at the DIC were convenient. DIC served as venues for meeting, social gathering and clinical activities [24]. A comprehensive package of services, such as HIV testing and counselling, condom distribution, ART initiation, referral for ART, and etc., were carried out by clinicians and lay health workers or peer educators in these centres. The DIC were linked to either a community-based ART centre or primary health centre (PHC) for technical support on all aspects of ART provision [17, 18, 24].

A DIC with outreach through a mobile team provides, in addition to DIC activities, a mobile health team comprising of clinicians, nurses and peer educators conducting ART outreach to venues of clients’ preference (the patient’s home, brothels, and PHC) for HIV testing, ART enrolment and initiation on specific days [21, 22, 24]. Home-based ART services were provided by the mobile health team in Tanzanian study [21]. Four studies, conducted in Tanzania, Zimbabwe and Nigeria, implemented DIC and mobile ART or outreaches for HIV testing services and/or ART initiation or refill [21–24]. In two studies, a mobile health team, comprising of clinicians, nurses and peer educators, were engaged to provide ART services through community-based HIV testing and counselling (CBHTC) and ART [21, 22]. Only one study offered home-based ART initiation [21]. The Nasarawa state study in Nigeria implemented a DIC and mobile ART outreach for MSM, FSW and PWID [22]. In the LINKAGE program, enhanced peer-led outreach was implemented for MSM and FSW in Ivory Coast. This strategy involved one-on-one meetings with KP to provide condoms and lubricants, information and education activities on HIV, and referral of peers for HIV testing and ART initiation. This enhanced peer outreach approach included intensive and targeted community-based activities aiming at improving HIV testing uptake and linkage to ART services [23, 24].

Six studies reported using community-based health centre strategy in delivering ART services to either FSW or MSM [19, 25–28]. These centres were dedicated to either MSM or FSW and were established by research institutions or NGOs to provide comprehensive HIV and STI services. Health care professionals (mostly clinicians) provided HIV care and treatment services to clients in these centres. Services provided in these centres included condom distribution, ART initiation, viral load testing, etc. Members of KP (such as key opinion leaders) and health care professionals together managed and coordinated activities in these community-based health centres. Four studies reported on findings from community-based health centres for MSM [19, 25–27] and one paper described care provided at a community-based health centre for FSW [28]. Community-based health centres were implemented in Kenya and Nigeria, where lay workers and KP peers supported and health care professionals provided ART care to HIV positive MSM [19, 26, 27]. One study described outcomes from a community-based health centre for FSWs in Cotonou, Benin [28].

#### Roles of KP Communities in CBART Service Delivery

All the community-based ART programs included in this review engaged KP or lay health workers to provide HIV care and/or ART (Table 3). They were involved in ART care to ensure sustainability and programme ownership by members of KP, and improve their access to quality HIV services.

#### Community Mobilisation (Peer Education and Navigation)

In all the KP-CBART programmes described in this review, FSW and MSM were involved as peer educators or mobilisers. They actively mobilised their peers and KP communities for HIV testing, linkage to HIV care, ART initiation and viral load testing. Services offered by trained peer educators include venue-based peer education, condom distribution, ART referral with or without assistance (escort services) and clients tracking for ART refill.

#### ART Service Delivery (ART Initiation, ART Refill, ART Adherence and Tracing of LTFU)

Members of the KP community, trained as peer educators, supported ART delivery in the community, through DIC, outreach venues (including home-based venues), and community-based health facilities. Eleven studies documented KP roles in antiretroviral therapy delivery, they offered ART referral to HIV positive patients and medication adherence counselling to those on ART [17–19, 21–27]. Furthermore, peer educators and navigators tracked patients who were LTFU in the project, either through SMS, phone calls or physical contact to improve retention in care.

In the DIC and mobile health team model in Tanzania, peer educators were reported to have initiated HIV-positive FSW on ART [21].

#### Training

In the Tanzania study, the KP-CBART delivery programme was designed in line with the national ART guidelines and the national community-based HIV and AIDS services guidelines [21]. Trainings that were conducted prior and during implementation included assessment of clients’ readiness and ART initiation, ART delivery, ART adherence and use of protocol on referral for advanced treatment in comprehensive treatment centres (Table 3). Meanwhile, in the MSM study in Nigeria, care providers received standard training on provision of culturally competent services (provider sensitivity training) and management of HIV/STD diagnosis [25, 26]. Furthermore, additional trainings and workshops organized for KP community workers and care providers included topics on community mobilisation strategies, HIV/STI prevention (condom negotiation, ART adherence), family planning, gender based violence (GBV), financial security, police sensitivity training and sex worker rights [17].

Moreover, trained KP offered psychological and social support through their leadership and membership in support groups such as Shikamana support group, Savings and Violence support groups, Adherence Sisters program in Tanzania and Zimbabwe [17, 18]. These support groups were coordinated by programme staff, peer educators and other key stakeholders.

KP were also involved in HIV programme planning, implementation and evaluation. In Tanzania, FSWs were members of the Community Advisory Board that offered guidance on KP-programme implementation in the study-region [17].

### Quantitative Data on KP-CBART Outcomes

#### HIV Testing Uptake

Across all studies, KP were tested, diagnosed with HIV and linked to HIV care (Table 4). HIV testing was strategic and targeted, therefore yielding high number of HIV positive results in the studies from Nigeria, Cote d’Ivoire, and Tanzania [17, 22, 24, 25]. One of the studies reported a lower rate HIV positive results in the community-based intervention arm compared to the control [17].

#### ART Uptake

Three articles compared a CBART intervention with a control arm for ART initiation among KP. In two studies conducted in the Tanzania and Zimbabwe, CBART was significantly associated with a higher likelihood of ART initiation. For one study the correlation was not significant (Table 4) [18]. Overall, between 54.7 and 100% of KP who tested HIV positive were initiated on ART in the community-based settings. Factors found to be associated with high ART uptake were a high level of education and having a strong social network [18].

#### Retention in Care

The definition of retention among those started on ART varied across programmes and depending on the study, retention in care was assessed at different timepoints (Table 8). Retention in care was operationalized in different ways across the included studies i.e. current on ART, completed 6 months of ART, and default on ART appointment of less than 2 months. The RCT study in Zimbabwe compared retention on ART (proportion of patients who are active on ART at 18 months) between those in a control arm and those in a CBART arm. Retention was significantly higher in the CBART arm (81.3% vs. 63.8%; p = 0.013) [17]. The Tanzania’s RCT and MSM study in Nigeria did not report retention outcomes. Retention in the intervention arms of the RCTs and prospective cohort studies was higher than 80% [17–19, 21]. One study compared the proportion on ART among all those who tested HIV positive, and did not find a difference between the control and the CBART arm (83.0% vs 86.3%, p = 0.22) [18]. Factors reported to be associated with attrition included low level of education, unemployment, stigma and lack of social network or support (Table 8). Other reasons for LTFU includes high mobility of sex workers when changing work environment and seeking clients in other cities or countries [28]. In the community-based HIV test and counselling (CBHTC) mobile and home-based ART study in Tanzania, there was no statistically significant difference for patient characteristics between those LTFU and those retained in care in the intervention arm (KP-CBART). However, in the control arm (facility-based care), clients who were LTFU, were younger and less likely to go to other regions for sex work in the 6 months before study assessment [18].

#### Viral Suppression

Eight papers reported on viral load suppression (VLS) (Table 7). KP who received ART in the community-based interventions achieved non-significantly higher VLS rate compared to the control arm [17, 18, 21] except in the Kenya study that reported sixfold higher odds of viral suppression (OR 6.24, 95% CI 1.28–30.5, Z = 2.26, p = 0.02) among gay, bisexual, and men who have sex with men (GBMSM) [19]. One of the studies found that having astronger supportive social network increased the odds of viral suppression [21].

## Discussion

Findings from this scoping review on the effect of KP-CBART on outcomes along the HIV care continuum ranging from HIV testing to viral load suppression and retention in care including ART delivery, showed that outcomes in KP-CBART were at least as good as in facility-based care. Our conclusion is coherent with the conclusion from a recent review on CBART for FSW, even though most of the included studies did not overlap, as different inclusion criteria were used [13]. Our review only included studies for which ART delivery was part of the intervention, and studies in which KP had a role in KP-CBART delivery. We used these criteria as community-based HIV services without activities that enhance linkage to ART do not always results in an adequate uptake of ART [29].

Our review showed that there are few community-based ART interventions targeting key populations and most were implemented at small scale and in research settings. Three approaches to KP-CBART were described, namely through (a) community drop-in-centres, (b) community drop-in-centres plus outreach through a mobile team or (c) community-based ART centres. What differentiates these models from each other are the locations of service delivery, the roles of care providers in ART delivery, and the package of HIV services offered to KP. From the present review it is not possible to infer which model works best for which population or setting. Already planned research of the existing KP-CBART programmes using realist evaluation will provide more evidence on the contextual factors and types of CBART model that favour good treatment outcomes.

CBART models were designed to meet the needs of KP communities, by attempting to reduce or dampen the effect of known socio-cultural and health system barriers to HIV prevention and treatment services [11]. The community drop-in-centre provides opportunities for members of KP to meet and give support to each other; and at the same time, to access HIV prevention and treatment services. Formation of support groups or buddy systems may be another critical element that contributed to engagement in care and medication adherence in the KP-CBART programmes [30]. For example in the Sisters programme, a trusted partner or sister is paired with a FSW, who is on ART or pre-exposure prophylaxis. These ‘partners’ act for each other as treatment buddies and they attend support group meetings together and remind one another of ART appointments [18]. The Shikamana project in Tanzania established Loans and Savings Support group to empower FSWs [17]. Through financial support and training, FSWs were empowered to take financial responsibility for their health (nutrition, transportation, and etc.). Moreover, KP were engaged in a large variety of tasks [17].

KP were trained as peer educators to support HIV service delivery [17, 19, 21, 24]. The involvement of KP as care providers in CBART may have facilitated HIV service delivery because they were able to understand the needs of the KP community better and their peers trust them more. With appropriate training and supervision, task-shifting of medical tasks such as ART refill is possible. Provision of peer-support and health navigation can improve engagement throughout the continuum of HIV care and treatment [31]. There is need to understand the causative mechanisms for observed outcomes in KP-CBART, as perceived by the participants, and the context conditions under which this works. How the involvement of KP in care provision was perceived by KP-CBART stakeholders has not been documented. Whether KP involvement indeed increases the acceptability of HIV service utilisation, needs to be assessed by future studies, as well as the extent to which KP can be involved in tasks traditionally performed by medical professionals.

Another hypothesis that needs further empirical testing in different settings is that the increase in HIV case identification in KP-CBART programmes/through the KP CBART service delivery model is probably due to sensitisation and activities of the outreach workers (clinical staff, peer educators) in the programme, illustrating the importance of providing a comprehensive package of services [32]. Recruitment into the CBART programme was respondent-driven (‘snowballing’), a process whereby peer educators were trained to identify and counsel KPs within their network and as such enhance HIV testing and ART enrolment [18, 21]. In the LINKAGES program, The KP-CBART (EPOA) engaged HIV-positive KPs as “peer mobilisers” in hotspots to mobilise and convince peers in their network for HIV testing and linkage to ART [23, 24]. This approach offers an opportunity for strategic HIV testing and could be leveraged to provide social network and index testing to KP. The high HIV positivity rates reported in this review are similar to findings from studies on social network and index testing [33]. Unlike the KP-CBART, most CBART models in the general population (GP-CBART) do not include HIV testing services, because only clinically stable HIV positive clients are enrolled into the models. Those who test positive to HIV in the KP-CBART, are either referred for ART or onsite ART initiation is offered depending on the type of model.

Where measured, linkage to HIV care and ART uptake also improved in the KP-CBART programmes compared to the baseline [17] and was better or similar to findings in the facility-based care [21]. The mobile health teams offer HIV counselling and testing, ART (both referral and initiation) in outreach venues or hotspots and home based platforms. In situations where ART initiation is not possible, potential clients were referred with an escort to a drop-in-centre with ART capacity or to a health facility for ART initiation.

ART uptake in the community-based health centre for MSM in the Nigeria study was remarkably lower (50.1–54.7%%) compared to the other two models [25, 26]. The difference in population type (MSM vs FSW) and the lower level of involvement of MSM in HIV service delivery may explain the low ART uptake in the community-based health centre model.

The rates of adherence to medication and retention in care (Tables 5 and 7) in CBART models are comparable to conventional HIV care (Table 7). Evidence [17] suggests that retention in care is correlated with the age of patients and level of education showing that there is need to address the social determinants of health among the KP to further improve their engagement in care.

Where measured, KP receiving ART through the CBART programme, achieved a similar viral suppression rate compared to those in the standard care model [17, 18]. However, viral suppression rate is still lower than the UNAIDS 95:95:95 target [34]. To improve access to viral load testing and increase viral suppression rates among KP, there is need to improve on logistics for viral load testing to reduce the turn-around time for viral load test result in the HIV programmes [35]. KP peers can assist with viral load sample collection and transfer from outreach venues, hotspots and drop-in-centres to PCR laboratories. If case management and medical tasks can effectively be shifted to community members such as trained KP, comprehensive KP-CBART programme may become more sustainable [16].

The major difference between CBART organised for PLHIV in the general population and KP-CBART is that the care package was more comprehensive in KP-CBART models, and that the role of lay health workers and peers was not limited to counselling and ART refill. In the trained KP-CBART service delivery model, trained peer educators participated in a wide range of activities, such as ART outreach (mobilising for HIV testing and ART), distribution of condoms, tracking LTFU, referral for ART initiation, and ART initiation. For example, in the Tanzania study, the peer educators and other members of the mobile health team initiated HIV positive FSW on ART. It was not clear for this intervention which type of training peer educators received and how much and what kind of support they received when initiating ART [21].

KP-CBART models can be adapted to lessons learnt from CBART for general population and vice versa. A review of CBART for the general population concluded that clinical outcomes for participants on treatment were at least comparable with facility-based ART and are likely to be cost-effective [36]. KP-CBART share some similarity with differentiated service delivery (DSD) models for the general population, howbeit a lot of lessons and best practices can be learnt from DSD models implementation in the general population (GP). DSD models among the GP include client-managed, health care worker-managed and out-of-facility (individual) models [37, 38]. Similarities and differences between KP-CBART models and CBART for the general population is shown in Supplementary Table 1).

When compared one-to-one with facility-based care in the RCT, outcomes in KP-CBART were at least as good. Compared to baseline, KP-CBART improved the continuum of HIV care and treatment outcomes across different settings in sub-Saharan Africa (Tanzania, Nigeria, Zimbabwe and Cote d’Ivoire) [17, 18, 21, 22, 24]. However, this does not imply that all HIV care should be community-based and by KP community members. CBART and conventional facility-based HIV care are likely to have complementary effects, as they meet the needs of different populations. Both approaches are needed to meet the UNAIDS 95–95–95 targets [1, 7]. KPs may access ART care in conventional health facilities and achieve optimal treatment outcomes if barriers to care are minimised. KP-CBART can complement facility-based care by offering ART services to those who cannot access care in the regular health facilities. Therefore, national HIV programmes and HIV programme managers should promote KP-friendly facility-based care alongside KP-CBART to maximize access to HIV care/tailor service delivery models according to actual needs and context.

The summary of strength and limitations of studies included in this review is presented in Table 9. Community-based ART interventions described in this review mainly apply to FSW. Six of the twelve reports described CBART programs for FSW, two reported data for both FSW and MSM, while four reports showed data from MSM. No study specifically target PWID and transgender people. This limits the generalisability of the findings from this review to specific KP subgroups. The heterogeneity of CBART interventions and study designs did not favour conducting a meta-analysis. With increasing evidence on KP-CBART, future research should consider a systematic review with metaanalysis to provide a more superior evidence on the models. While acknowledging these limitations of our study, a strength of this review was the systematic approach to the review following the PRISMA ScR guidelines. The evidence reported here seems robust, as findings from both randomised and observational studies showed similar effects of KP-CBART on outcomes along the HIV care continuum. Moreover, the observational studies (non-experimental design) of KP-CBART offer evidence from real-life situations that could inform adaptation and scale-up of similar KP-CBART initiatives in sub-Saharan Africa. This review revealed lack of data on contextual factors and mechanisms that trigger observed outcomes. Therefore further operational research is recommended to better understand the effect of contextual conditions on the implementation of CBART for KP in real world settings, and how KP-CBART may be best adapted to fit the specific needs of clients.

**Table 9:** Summary of strength and limitations of studies included in the review

Study	Strength	Limitations
Kerrigan et al. 2019 [17]	Cluster randomised controlled study [2 clusters, randomized by community]	Limited ability to draw inferences to wider FSW population • Small sample size • Limited number of communities Observation bias—no blinding • Cohort effect: monthly contact with participants to ensure retention
Cowan et al. 2018 [18]	Cluster randomised controlled study (14 clusters, randomized by sites) An integrated and prospective assessment was done alongside the trial to better understand strengths and weaknesses of the programme implementation	Limitations to assessment of causality: • Not all potentially confounding factors were overcome by randomization: other interventions (such as microplanning) are linked to outcomes were more frequent in the intervention arm as compared to the control group • Short duration of intervention makes it difficult to demonstrate effect at the population level • Randomization at community level: secondary outcomes are dependent on the characteristics of participants in the community, and communities differed Selection bias during enrolment- respondent driven sampling through snowballing (difficulty in determining the refusal rates) Cross-over effect: intervention could change the network structures in the control group
W. Tun et al. 2019 [21]	Prospective cohort study: good data Real world setting • Transferability of findings • May inform scale-up	One region had the intervention, one region acted as control group. The intervention was not randomly assigned, which reduced the comparability of study arms (confounding bias may have occurred) Observation/information bias • Treatment outcomes were self-reported (social desirability bias may have occured)
Ibiloye et al. 2018 [22]	Real-world setting	Short study period Transferability of findings • Data were collected from a single setting Retrospective study design may have caused selection bias
Olawore et al. 2020 [23]	Real-world settings	• Use of program data with inherent data inconsistencies and missing data • Monetary incentives were given and could have introduced participation bias • Recall and social desirability biases
LINKAGES 2017 [24]	Real-world setting Big data (country-wide data)	Report is based on program data (missing data) Report describes % contribution of intervention to ART care cascades in the program, does not show the actual numbers
Ramadhani et al. 2018 [25]	Well established cohort	Attrition bias: High LTFU among study participants, possibly causing selection bias Possible re-structuring of patients’ social network following HIV diagnosis
Graham et al. 2018 [19]	Randomised controlled trial	Study was conducted in a controlled environment, thus may not reflect reality in low resourced setting Follow-up was limited to 6 months Results cannot be generalized to the broader population of Kenyan GBMSM because men who participated in this study may differ from other HIV-positive GBMSM in Kenya
Kayode et al. 2020 [26]	Prospective cohort, thus reducing incomplete data	Under/over estimate of overall LTFU due to silent transfer (inability to assess whether lost participants re-engaged in care) Study was conducted in two cities and may not be generalizable to other areas in Nigeria
Charurat et al. 2015 [27]	Prospective cohort	Selection bias from respondent driven sampling
Napierala et al. 2018 [20]	Large cohort	Selection bias as a result of respondent driven sampling Reporting bias: self report of HIV status
Diallo et al. 2020 [28]	Prospective cohort	Reduction in the power of the study due to high rate of participants’ drop outs from the study Self-reporting of medication adherence is limited by social desirability and recall bias

## Conclusions

The results from the various studies in sub-Saharan Africa showed the potential of community-based ART delivery to improve engagement in HIV care and ART related outcomes among KP in Africa. Across studies that compared KP-CBART with facility-based care, outcomes in terms of ART uptake, adherence to ART, retention in care and viral suppression were at least as good in KP-CBART as those obtained for KP in facility-based care. Therefore, to fast track the achievement of the UNAIDS 95–95–95 target by 2030 in sub-Saharan Africa, national programmes should promote policies/develop/pilot guidelines on task shifting of medical tasks to members of KP and lay healthcare workers to enrol additional KP in care, providing an alternative model to facility-based care. When community-based ART service delivery for KP would be scaled up to complement facility-based care, future research should focus on long-term benefits of community-based care and explore views, experiences, and preferences of different stakeholders with regards to different community and facility-based models of care, and the mix of differentiated service delivery models in different settings.

## Supplementary Information

Below is the link to the electronic supplementary material.

Supplementary file1 (DOCX 15 kb)

Supplementary file2 (DOCX 13 kb)

## Data Availability

Not applicable.
