# Supplementary material for: Community-Based ART Service Delivery for Key Populations in Sub-Saharan Africa: Scoping Review of Outcomes Along the Continuum of HIV Care
Source: AIDS Behav. 2022 Jan 17;26(7):2314–37. doi: 10.1007/s10461-021-03568-3 (PMC9162992; doi:10.1007/s10461-021-03568-3)
Supplement: Supplementary file 1 — Supplementary file1 (DOCX 15 kb) [file 10461_2021_3568_MOESM1_ESM.docx]

*Supplementary table 1. Comparison between community-based ART for KP and general population*

| **Service delivery models** | **KP-CBART** | ***CBART in the general population** | | |
| --- | --- | --- | --- | --- |
|  |  | **Out-of facility Individual** | **Client-managed groups** | **Health care worker-managed group** |
| Examples of model | Drop-in-centre, drop-in-centre plus mobile team, community health centre | Mobile outreach, fixed community ART distribution points, community pharmacy, home delivery | Community ART group (CAG), community adherence group, peer support group (PSG) | ART adherence clubs, youth clubs and patient adherence groups |
| Who is eligible | KPLHIV ***(clinical stability is not a requirement)*** | On ART for 12-month, clinically stable, undetectable VL | Stable clients (6 months on ART) | Stable clients |
| **ART refills**  What | ART refill, adherence support, referral for ART, weighing, symptoms screen, | ART refill, Adherence support, referral if unwell | ART refill, Group adherence support | ART refill, brief symptom screen, adherence support, weight measurement, lab tests |
| Where | Community venue (outreach venue, hotspots), home | community venue, home | Community venue, home | Community venue, Primary health clinic |
| When | Monthly | 1-3 months | 1-3 months | 2-3 months |
| Who | Peer/volunteer, lay health care workers | lay health care worker, peer/volunteer | client, peer | Lay HCW |
| **Package of services** | More comprehensive: sensitization, HIV prevention, care, and treatment, STI management | Mainly antiretroviral therapy: ART refill and adherence support | | |
| **Clinical consultation**  What | lab. Test, clinical monitoring, ART refill, ART rescripting | lab. Test, clinical monitoring, ART refill, ART rescripting | lab. Test, clinical monitoring, ART refill, ART rescripting | ART rescripting, clinical monitoring, ART refill |
| Where | DIC, PHC and community health centre | community venue, primary health care | primary care clinic | Primary care clinic |
| When | 1 – 2 months | 6-12 months | 6 months | Annual |
| Who | Doctor, Nurse | Nurse | Physician, nurse | Physician, nurse |

**synthesised from https://www.differentiatedservicedelivery.org/*

*DIC- drop-in centre, PHC-primary health centre, ART-antiretroviral therapy, KP- key populations, CBART-community-based ART, KP-CBART, community-based ART for key populations, HCW- health care workers, KPLHIV-key population living with HIV*
