# Supplementary material for: Community-Based ART Service Delivery for Key Populations in Sub-Saharan Africa: Scoping Review of Outcomes Along the Continuum of HIV Care
Source: AIDS Behav. 2022 Jan 17;26(7):2314–37. doi: 10.1007/s10461-021-03568-3 (PMC9162992; doi:10.1007/s10461-021-03568-3)
Supplement: Supplementary file 2 — Supplementary file2 (DOCX 13 kb) [file 10461_2021_3568_MOESM2_ESM.docx]

**Supplementary Table 2: Search string for Web of Science**

|  | **Search string for Pubmed** |
| --- | --- |
| **Population** | (((ALL=(“sex workers” OR “FSW” OR ‘’female sex workers’’ OR “MSM” OR “Men who have sex with men” OR “gay men” OR “transgender” OR “TG” OR “persons who inject drugs” OR “PWID” OR “ivds” OR ‘’key population*’’)) AND AB=(“Africa” OR “sub Saharan Africa’’ OR “Algeria” OR “Angola’’ OR ‘’Benin’’ OR ‘’Botswana’’ OR “Burkina Faso” OR ‘’Burundi’’ OR “Cape Verde” OR ‘’Cameroon’’ OR “Central African Republic” OR ‘’Chad’’ OR ‘’Congo’’ OR “Costa Rica” OR “Côte d’Ivoire” OR “Ivory Coast” OR Djibouti OR Egypt OR Eritrea OR Ethiopia OR “Equatorial Guinea” OR ‘’Gabon’’ OR ‘’Gambia’’ OR ‘’Ghana’’ OR ‘’Guinea’’ OR “Guinea Bissau” OR “Ivory Coast” OR ‘’Kenya’’ OR ‘’Lesotho’’ OR ‘’Liberia’’ OR ‘’Madagascar’’ OR ‘’Malawi’’ OR ‘’Mali’’ OR ‘’Mozambique’’ OR ‘’Namibia’’ OR ‘’Niger’’ OR ‘’Nigeria’’ OR ‘’Papua New’’ OR ‘’Rwanda’’ OR ‘’Ruanda’’ OR ‘’Senegal’’ OR “Sierra Leone” OR ‘’Somalia’’ OR “South Africa” OR “South Sudan” OR ‘’Sudan’’ OR ‘’Tanzania’’ OR ‘’Togo’’ OR ‘’Uganda’’ OR ‘’Zambia’’ OR ‘’Zimbabwe’’) |
| **Intervention** | AND ALL=(“Community” OR “peer” OR “home” AND “HIV” OR “AIDS” OR “antiretroviral therapy” OR “ART” OR “HAART” OR “acquired immunodeficiency syndrome”)) |
| **Comparison** | Not applicable |
| **Outcomes** | AND ALL=(“linkage” OR “retention” OR “attrition” OR “viral suppression” OR “treatment outcome” )) |
